# A Rare Case of Insular Epilepsy: Not To Be Missed in Refractory Epilepsy Patients

**DOI:** 10.7759/cureus.5434

**Published:** 2019-08-20

**Authors:** Mohan Kurukumbi, James Leiphart, Lillian Singer

**Affiliations:** 1 Neurology, Inova Health System, Fairfax, USA; 2 Neurosurgery, Inova Neuroscience Institute, Falls Church, USA; 3 Adult Neurology, Inova Fairfax Hospital, Falls Church, USA

**Keywords:** insular epilepsy, refractory epilepsy, insular resection

## Abstract

Insular epilepsy often goes under-recognized and misdiagnosed due to the similarity of its features with temporal lobe epilepsy and the common exclusion of the insula during intracranial electroencephalography (iEEG). Here, we present a case of medically refractory epilepsy in a 43-year-old male with a 12-year history of tonic-clonic seizures. Insular epilepsy cases are often considered for diagnosis in the setting of abnormal insular pathology, such as a low-grade central nervous system (CNS) lesion. This is a unique case of non-lesional insular epilepsy, successfully managed by the resection of the insular cortex.

## Introduction

Insular epilepsy is a rare condition in which the insula is the primary or one of two principal epileptogenic zones. Its semiology resembles that of temporal, parietal, and frontal lobe epilepsies, and thus it often goes misdiagnosed and undertreated [1]. Management with surgical resection of the insular cortex has been shown to increase the likelihood of lifelong seizure freedom, so recognition of insular involvement by intracranial electroencephalography (iEEG) or stereo-electroencephalography (sEEG) is key to providing optimal care [2]. Here, we present a case of medically refractory insular epilepsy, discovered on iEEG and responsive to insular cortex resection.

## Case presentation

A 43-year-old Caucasian male with a past medical history of hypertension and 12 years of intractable seizures was evaluated for craniotomy and iEEG monitoring to localize and resect seizure foci. Seizure semiology included the patient awakening from sleep, bilateral hand and oral automatisms, head turning toward the left, blinking, and left lower extremity tonic posturing. The patient was completely amnestic regarding these events. Owing to intractable seizures, neurological assessments had revealed worsening neurocognitive decline, which created a poorer quality of life. His failed anti-seizure medications included clobazam, lacosamide, and lamotrigine, with persistent breakthrough seizures one to three times per month.

Previous electrocorticography (ECoG) had captured right frontal and right parietal onset seizures, but no depth insular or hippocampal electrodes were placed during this recording. Magnetic resonance imaging (MRI), functional MRI (fMRI), and positron emission tomography (PET) scans were done. The PET scan showed reduced uptake in the right hemisphere and right basal ganglia. fMRI showed a language predominance in the left hemisphere. Brain MRI showed post-craniotomy changes over the right parietal region, without evident parenchymal lesions.

The patient was status-post vagus nerve stimulation (VNS) implantation, video-EEG (vEEG), and a prior right-sided craniotomy and grid placement for an intracranial electrodes study. vEEG monitoring revealed tonic-clonic seizures with loss of consciousness. The patient described aura preceding seizure activity as being characteristically “sick - not right feeling.” The initial iEEG study revealed that seizure foci were at the edge of the grid and did not confirm the localization of seizure foci clearly. As the previous iEEG was non-confirmatory, and the patient continued to have recurrent, disabling seizures, current iEEG was considered. Depth electrodes were placed in the right hippocampus and insula (anterior and posterior), in addition to an 8x8 grid over the neocortex.

**Figure 1:**
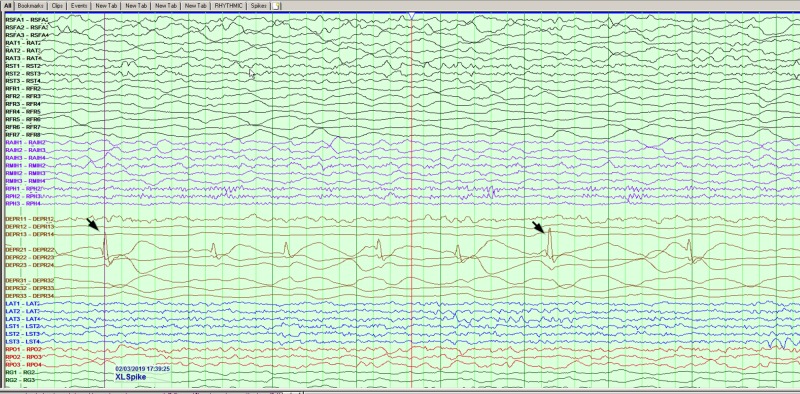
Interictal sharp waves noted from depth electrodes at the right anterior insular region

**Figure 2:**
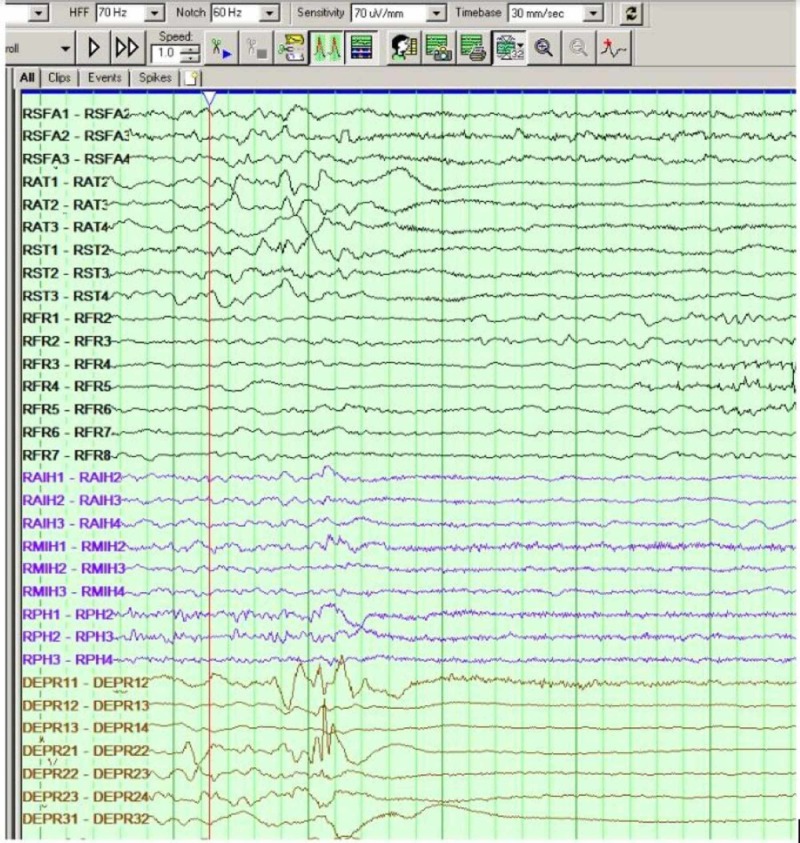
Seizure clinical and electrographic onset at the right insular depth electrode

**Figure 3:**
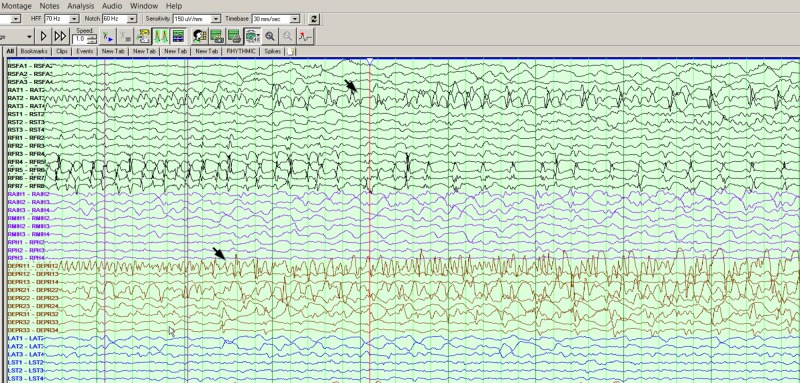
Secondary spread to the right hippocampal and right anterior temporal region

ECoG done during the operation confirmed that seizures were originating from the right anterior insular cortex and rapidly spreading to the right frontal and temporal operculum (Figures 1-3).

The patient subsequently underwent a right frontotemporal insula lobectomy without serious complications, except minimal left-sided weakness. The patient was monitored in the intensive care unit (ICU) following surgery, demonstrated improvement in participation and alertness, and reported an improvement in left-sided weakness secondary to right-sided surgery. He has remained on his medication regimen and seen occupational therapy/physical therapy (OT/PT) to improve weakness. At six months post-surgery, the patient reported no seizures and noted an improvement in his left-sided weakness, approaching his near-baseline level.

## Discussion

The insular cortices are situated at the bottom of the lateral cerebral fossa bilaterally, deep within the lateral sulcus, which separates the frontal and parietal lobes from the temporal lobe [3]. The deep positioning of the insular cortex obscures it from the surface view of the brain.

The insula is further divided by the central insular sulcus into anterior and posterior lobes, which are distinct in their roles [4]. The anterior lobe is involved in regulating cognitive control processes, affective processes, emotional awareness, and empathy [5]. The posterior lobe plays a role in vestibular perception, sensorimotor processing, pain encoding, temperature perception, and thermoregulation [6-7]. The insula also has an autonomic role. By surveilling the cardiac responses to internal and external stimuli, the insular cortex integrates limbic features like memory and cognitive constructs into the physiologic response, in order to maintain homeostasis [8]. Damage to the insula has been linked to impaired cardiac contractility and even sudden cardiac death [8]. The insula also plays a key role in the perception of dyspnea; damage to the insular cortex has been demonstrated to reduce the feeling of shortness of breath, which poses a threat to the maintenance of homeostasis in circumstances begetting respiratory distress [9].

Although the exact incidence of insular epilepsy is unknown, the insula as a primary epileptogenic zone is uncommon. In a study of 50 patients with atypical TLE, sEEG demonstrated that, of the 86% of seizures that spread to the insula, only 12% actually arose from it [10].

The differing functions of the anterior and posterior lobes of the insula also suggest that seizures originating from either lobe will produce distinct symptomatology. Cortical stimulation of the anterior lobe characteristically produces viscerosensory symptoms, in contrast to the somatosensory symptoms produced by the stimulation of the posterior insula [4]. The ictal sequence for insular epilepsy has been described as follows: in the setting of full consciousness, there is an initial sensation of laryngeal constriction and large swaths of cutaneous paresthesias. This typically precedes a complex partial seizure and is followed by dysarthria and convulsive symptoms [10].

The insula is often overlooked as a primary focus for medically refractory epilepsy. Insular epilepsy is often concurrent and confused with perisylvian seizures, temporal plus epilepsy, hypermotor seizures, MRI-negative frontal and parietal lobe epilepsies, and insular lesions [1]. However, the localization of seizure activity to the insular cortex and subsequent resection can be a definitive cure, so fastidious monitoring by sEEG or iEEG is critical.

Insular epilepsy and temporal lobe epilepsy

Insular epilepsy has symptomatology that distinguishes it from TLE, PLE, and FLE and should be explored as an etiology of seizure activity in the following scenarios: TLE-like patients exhibiting laryngopharyngeal discomfort, throat constriction, limb paresthesias, thrashing, or pedaling behaviors; PLE-like patients with oral or wide cutaneous paresthesias; and FLE-like patients with somatosensory aura preceding nocturnal hypermotor seizures [4].

Our patient’s seizures typically occurred during sleep, manifesting as head turning to the left and perioral automatisms, which is usually characteristic of temporal lobe seizures. The insular focus was thus overlooked and was not considered in the previous ECoG. This led to delayed localization in this patient.

Insular epilepsy should be considered in any patient with intractable seizures for whom TLE surgery has failed; as it stands, seizure freedom rates after TLE surgery are less than 70% at the three-year follow-up [8]. This suggests that more cases of TLE should be classified as “temporal plus,” with an investigation into insular involvement. Ascertaining whether a patient has "temporal plus" epilepsy is important, as studies have found these patients have reduced seizure freedom postoperatively as compared to those with pure TLE [8].

Typical association with CNS lesions

Insular epilepsy is often found in the setting of low-grade CNS tumors in or affecting the insula, most commonly gliomas [4]. The absence of a CNS lesion on imaging should not rule out insular involvement in refractory epilepsy; indeed, our patient’s case highlights the necessity of thorough electrophysiologic investigation, even when an insular lesion has been ruled out.

Methods for detection

Scalp EEG does not suffice for the detection of insular involvement, due to its limited coverage: insular epilepsy must be confirmed by intracranial ictal recordings [4]. It is particularly difficult to localize seizure activity to the insula using electrophysiology, given that it lies deep to the frontal and temporal lobes [11]. iEEG is currently accepted as the most effective diagnostic modality for identifying insular epilepsy [12]; our patient’s lack of morbidity is one testament to its efficacy and safety.

Recommended treatment and complications

Insular resection is an appropriate treatment for medically refractory epilepsy localized to the insula or “temporal plus” epilepsy involving the insula [4]. One study demonstrated 83% seizure freedom at the 36-month follow-up, after complete insular resection [2], though this was limited by a small sample size of six. Other studies suggest that insular resection only improves the likelihood of lifelong seizure freedom in cases of temporo-insular epilepsy, not solely insular epilepsy [8]. In this case, evidence of temporal involvement should be required to indicate insular resection.

The greatest complication associated with insular resection is an aphasic disturbance, though the role of the insula in speech processing is unclear [4]. Another study has shown that insular resection patients had poorer facial recognition abilities and diminished capacity for empathy postoperatively [13]. Our patient reported contralateral weakness following insular resection but has improved over time and with the help of OT/PT.

## Conclusions

This case is reported due to the common oversight of insular involvement in refractory epilepsy. iEEG monitoring often excludes the insula, delaying its recognition as a seizure focus; however, early identification has the potential to limit epilepsy morbidity and prompt action that may eradicate further seizure activity. Surgical resection has the potential to be a definitive treatment, as demonstrated by our patient’s continued seizure freedom.
